# 

**Published:** 1867-01

**Authors:** 


### INDEX OF HALL'S JOURNAL OF HEALTH.

VOL. XIV, 1867

| | PAGE. |
|---|---|
| Air and Health | 8 |
| Adulterating Food | 59 |
| Army Sickness | 65 |
| American Manners | 88 |
| " Climate | 93 |
| Acidity | 17 |
| Autumnal Diseases | 179 |
| Angel's Wings | 214 |
| A Noble Fruit | 220 |
| Animal Grafting | 248 |
| Bronchitis | 1 |
| " What is it? | 2 |
| " Symptoms | 3 |
| Beef Tea | 16 |
| Bodily Strength | 46 |
| Bread Making | 73 |
| Bilousness | 76 |
| Bible and Business | 215, 157, 99 |
| Business Rules | 165 |
| Bachelors | 109 |
| Brain Work | 170 |
| Best Recommendation | 172 |
| Business, Getting into | 172 |
| Beverages | 189 |
| Beautiful Death | 212 |
| Brave Women | 214 |
| Bliss of Dying | 216 |
| Buried Alive | 219 |
| Blot, Professor, on Cooking | 244 |
| Burns Cured | 254 |
| Consumption | 63, 2 |
| " Symptoms | 67, 3 |
| Croup | 34, 16 |
| Cleanliness | 58 |
| Checking Perspiration | 75 |
| Clothing, Changing | 78 |
| Catching Cold | 85 |
| Common Sense | 101 |
| Contradictions | 104 |
| Chicken Pox | 108 |
| Country Summerings | 109 |
| Children Punished | 120 |
| Charities | 123 |
| Chimney Smoking | 167 |
| Cellars | 171 |
| Crazy Farmers | 177 |
| Cold in the Head | 183 |
| Cereal Food | 187 |
| Cancer of Liver | 218 |
| Cookery | 243 |
| Cook, Characteristics of | 245 |
| Clarifying Water | 247 |
| Cuts of Flesh Cured | 249 |
| Cookery | 243 |
| Chapping of Hands Cured | 260 |
| Cold Feet | 259 |
| Corns | 261 |
| Dry Soles | 255 |
| Diet for Invalids | 16 |
| Diptheria | 28 |
| Dentistry | 47 |
| Drink a Despotism | 66 |
| Drunkenness | 223 |
| Diarrhœa | 86 |
| Death, Beautiful | 212 |
| Dying Sensations | 220 |
| Driving Nails | 248 |
| Daughters Lost | 223 |
| Dead Wife | |
| Experimenting | 57 |
| Erect Position | 87 |
| Early Rising | 88 |
| Equinimity and Longevity | 251 |
| Fever and Ague | 28 |
| Frenzy | 154 |
| Farmers, Crazy | 177 |
| Forbearance | 201 |
| Forbodings | 218 |
| Feet Management | 258 |
| French Cookery | 244 |
| Fish as Food | 246 |
| Flesh Uniting | 249 |
| Frosty Weather | 250 |
| Food and Physic | 255 |

| | PAGE. |
|---|---|
| Feet Protected | 253 |
| Fear of Death | 221 |
| French Cookery | 244 |
| Fish as Food | 246 |
| Flesh Wounds | 249 |
| Getting up Wrong | 106 |
| Giving Advice | 174 |
| Glycerine Nutritious | 254 |
| Grafting in Animals | 248 |
| Health Secrets | 61 |
| Habit | 87 |
| Harsh Teachers | 147 |
| Hemorrhage | 169 |
| Hair Removed | 250 |
| Horseback Exercise | 253 |
| Intermarriage | 61 |
| Ice-Pitchers | 129 |
| Insaniiy Treated | 208 |
| Ill-Smelling Feet | 259 |
| Joking C ergymen | 91 |
| Lung Measure | 37 |
| Last Hope | 64 |
| Life's Battle | 106 |
| Lesson to Parents | 142 |
| Lending Money | 159 |
| Longevity | 166 |
| Life a Pupilage | 212 |
| Life Lengthened | 250 |
| Longevity and Equinimity | 241 |
| Mad-Stone | 29 |
| Madness | 48 |
| Malaria and Miasm | 103 |
| Married Life | 166, 104 |
| Mother's Remorse | 140 |
| Memory | 217 |
| Mineral Waters. | 256 |
| Norrow House | 232 |
| Old Man's Story | 148 |
| Private Things | 31 |
| Passing Away | 62 |
| Perspiration Checked | 75 |
| Pitching | 94 |
| Public Schools | 122 |
| Parental Corrections | 126 |
| Pio Nono's Health | 210 |
| Presidents' Testimonies | 194 |
| Passionate Outbursts | 251 |
| Recreation | 61 |
| Rest | 190 |
| Rational Deductions | 241 |
| Starvation | 7 |
| Skating | 30 |
| Sleeping Rooms | 61 |
| Spring Dangers | 74 |
| Sitting Erect | 87 |
| Sick-Room | 89 |
| Suspiciousness | 95 |
| Smoky Chimneys | 167 |
| Soups | 246 |
| Sores, Foul | 250 |
| " Offensive | 249 |
| " Painful | 255 |
| Sea-Sickness | 251 |
| Sea-Voyages | 252 |
| Skin, Blistered | 255 |
| Skin, Made Soft | 261 |
| Shoes | 257 |
| Throat-Ail | 2 |
| Tonsils Cut | 9 |
| Two Pictures] | 48 |
| Toe Nails, Ingrowing | 257 |
| Tomatoes | 60 |
| Traveling | 109 |
| Teeth Preserved | 187 |
| Two Meals a Day | 211 |
| Vital Capacity | 49 |
| Women, Intemperate | 29 |
| Women | 105 |
| Washing | 181 |
| Women Doctors | 242 |
| Water Clarified | 242 |
| Young Men | 177 |

